# Data Pre-Processing for Label-Free Multiple Reaction Monitoring (MRM) Experiments

**DOI:** 10.3390/biology3020383

**Published:** 2014-06-05

**Authors:** Lisa M. Chung, Christopher M. Colangelo, Hongyu Zhao

**Affiliations:** 1 Department of Biostatistics, Yale School of Public Health, New Haven, CT 06520, USA; E-Mail: lisa.chung@yale.edu; 2 W.M. Keck Foundation Biotechnology Resource Laboratory, Yale School of Medicine, New Haven, CT 06510, USA; E-Mail: christopher.colangelo@yale.edu

**Keywords:** multiple reaction monitoring, label-free, quality assessment, data normalization, proteomics, peptide, transition

## Abstract

Multiple Reaction Monitoring (MRM) conducted on a triple quadrupole mass spectrometer allows researchers to quantify the expression levels of a set of target proteins. Each protein is often characterized by several unique peptides that can be detected by monitoring predetermined fragment ions, called transitions, for each peptide. Concatenating large numbers of MRM transitions into a single assay enables simultaneous quantification of hundreds of peptides and proteins. In recognition of the important role that MRM can play in hypothesis-driven research and its increasing impact on clinical proteomics, targeted proteomics such as MRM was recently selected as the Nature Method of the Year. However, there are many challenges in MRM applications, especially data pre‑processing where many steps still rely on manual inspection of each observation in practice. In this paper, we discuss an analysis pipeline to automate MRM data pre‑processing. This pipeline includes data quality assessment across replicated samples, outlier detection, identification of inaccurate transitions, and data normalization. We demonstrate the utility of our pipeline through its applications to several real MRM data sets.

## 1. Introduction

Quantitative proteomics technologies estimate protein expression levels in a biological sample by measuring peptide abundance produced from each protein after trypsin digestion. They are commonly used to identify differentially expressed proteins between groups of samples, such as between diseased and normal samples or between wild-type and knock-out animals. They can also identify different levels of protein post-translational modifications, e.g., phosphorylation. Multiple approaches using mass spectrometry (MS) have been developed including stable isotope labeling by amino acids in cell culture (SILAC) [1], isobaric tagging technology for relative and absolute quantitation (iTRAQ) [2], and LC-MS/MS label-free quantification [3]. In SILAC and iTRAQ, peptides from different samples are labeled with different tags, mixed together and then simultaneously monitored by MS instruments. These methods often require large sample quantities and may be prone to labeling bias. Additionally, proteins identified and quantified are variable across replicated experiments because these approaches rely on stochastic precursor-ion selection [4]. The same peptide is often only observed in a fraction of the samples, leading to significant missing data problem. On the other hand, label free quantitation requires longer MS instrument times and large numbers of technical replicates for accurate chromatography as well as chromatogram realignment after data collection. Due to the complexity in whole proteome analysis and stochastic nature of these approaches, targeted protein analysis that detects and quantifies pre-selected components in a complex matrix with high sensitivity has emerged as an attractive alternative. It is particularly useful for clinicians aiming to identify biomarkers for diagnosis and personalized treatment of many human diseases.

Multiple reaction monitoring (MRM) is a targeted proteomics approach that quantifies protein abundance through a pre-defined set of precursor/fragment ion pairs from proteins of interest. Intensities of the ion pairs are measured by using a triple quadrupole mass spectrometer which consists of three quadrupole mass analyzers in series. The first and third quadrupoles serve as mass filters to select precursor and fragment ions with the specific mass-to-charge (m/z) values, respectively. The second quadrupole is used to collide a precursor into several fragments. A pair of precursor and its particular fragment ion is called a transition. For precise quantification of proteins, peptides, or peptide modifications, researchers have utilized stable isotope standards (SIS) for each ion pair. The stable isotopes can be generated by synthesizing stable isotope peptides (AQUA peptides [5]) or full length isotope labeled proteins (PSAQ [6]). This method has become increasingly popular due to its high accuracy and reproducibility [7,8,9,10]. However, the labeled standards can be costly and have to be prepared for each endogenous transition to monitor. It also doubles the number of ion pairs to be monitored. Label-free MRM quantification is an alternative method that extracts ion intensities from LC-MRM chromatogram with no or few standard isotopes. Recent studies suggest that label-free MRM quantification has high quality in measuring protein abundance of biological samples [11].

Although MRM is a very promising proteomics technology in biomedical research, it does pose many statistical and computational challenges. Similar across the analysis of all genomics and proteomics data, quality assessment and data normalization are critical pre-processing steps to ensure the data are properly processed so that the downstream analysis, such as abundance estimation and differential expression analysis, can be appropriately carried out. In this paper, we focus on MRM pre‑processing, a key step in MRM data analysis. Appropriate quality assessment enables researchers to detect and remove inconsistent transitions or problematic peptides as well as outlying runs. Many statistical approaches employed for genomics data such as microarrays are directly applicable to proteomics data sets. For example, hierarchical clustering can be used to cluster samples according to their overall similarities and to identify outlying samples. However, MRM data have some distinct data structures and unique characteristics that need to be considered. MS/MS peak properties including a retention time or background noise are important quality measures. Moreover, peak intensities from the same peptide should be consistent with the peptide collision pattern. In recent years, a number of research groups have developed and implemented several computational and statistical approaches for appropriately analyzing and interpreting proteomics data. Skyline performs peak detection and quantification and provides quality measure [12]. It evaluates background noise level for each quantified MS/MS peak and calculates a dot-product between quantified peak areas of the same precursor and their spectral library intensities. The value of this dot-product is then used to assess the quality of each peptide. However, Skyline only assesses the transition group quality within each run and highly relies on the MRM library information. AuDIT is an automated quality assessment tool for isotope labeled MRM experiments aiming to detect any inconsistent transitions within each peptide [13]. It assumes that the area ratio between a pair of endogenous transitions should be consistent with the ratio between a pair of corresponding SIS peptides. Thus, it is only applicable to labeled MRM experiments. Furthermore, it assumes that the heavy labeled transition should provide accurate quantification and no interference in sample matrix. mProphet classifies peptides into two groups according to their quality [14]. It utilizes linear discriminant analysis in a semi-supervised learning framework by utilizing decoy transitions as negative controls. A set of decoy transitions is constructed by reversing the peptide sequence or adding a small number to Q3 mass of each transition. Similar to the dot-product used in Skyline, the mProphet procedure is applied separately to each run, and may have limited performance with an increasing number of transitions to be monitored. Moreover, the result may not be reliable if some decoy transitions may in fact exist in the protein sample.

After data quality assessment of the peptides, data normalization is the next critical step in data pre‑processing [15,16,17,18]. An effective normalization method aims to minimize non-biological variations across samples and make the samples comparable. Such unwanted variation [19] can be introduced *via* instrumental variation during data acquisition, differences in the total protein amount loaded into the instrument, or sample preparation/processing variation. However, data normalization has not been well studied for MRM experiments. Several methods have been suggested, such as matching the median of overall peak intensities across samples [20], or adjusting the median of peak intensities from house-keeping proteins or internal standard peptides [21]. However, the performances and advantages of various normalization methods have not yet been widely discussed.

This paper is organized as follows. In Section 2, we introduce three example data sets that will be used to illustrate the need for quality assessment and normalization methods. Section 3.1 discusses several methods to evaluate peak, transition, and sample quality by utilizing retention time deviation and a robust linear regression model. Applications of various normalization methods and their performance evaluations are shown in Section 3.2.

## 2. Data Sets

### 2.1. Data Set 1: Rat Brian Post Synaptic Density

The postsynaptic density (PSD) is a specialized protein complex at the junction between two neurons. The PSD is enriched with protein components including neurotransmitters, cytoskeletal proteins, and signaling molecules. Expression levels of such proteins can be altered by drug response. Colangelo *et al.* identified around 1200 proteins in the PSD fraction from a discovery study on a Triple TOF 5600 MS (AB Sciex, Framingham, MA, USA) and developed an MRM-based protein quantification approach to understand synaptic activity [22,23]. In their study, the abundance of 112 selected target proteins was measured by 337 peptides and 1697 fragments (~3 peptides per protein, ~5 fragments per peptide). PSD tissues were collected from the brain region of 6 male Spradue Dawley rats (Charles River Laboratories, Wilmington, MA, USA) using a Percoll gradient method (GE Healthcare, Waukesha, WI, USA). For each run, 1 μg of digested peptides were injected and analyzed by 5500 QTRAP mass spectrometer. Each biological sample was run three times by randomization to assess the technical variability. Fragment-level peak intensities were detected and quantified by the SignalFinder™ 2 algorithm [24]. Various peak scores were recorded, including peak area, height, noise, and retention time. We use peak area as an intensity measure after log2 transformation. The full data set was uploaded at Yale Protein Expression Database [25,26] and publicly available [27].

### 2.2. Data Set 2: Cysteine String Protein

Cysteine string protein α (CSPα) is a presynaptic vesicle protein. It plays an essential role in the release of neurotransmitters required for synaptic maintenance. Deletion of CSPα in mice is known to cause synapse loss, neurodegeneration, and early lethality [28,29]. Zhang *et al.* [30] designed a study to compare the intensity profiles between CSPα knockout (KO) mice and wild type (WT). Brains from CSP-/- and WT mice were fractionated into synaptic cytosol fraction 2 (LS2), synaptic vesicle (LP2), and synaptic plasma membrane (SP). The MRM assay monitored 1151 fragments from 231 peptides (4–5 fragments/peptide) corresponding to 24 proteins (3–30 peptides per protein). Samples from one KO and one WT mouse were run with three technical replicates. All analyses were carried out on a 5500 Q-TRAP instrument coupled to an online Waters nanoACQUITY Ultra High Pressure Liquid Chromatography system. Data were initially processed using MRMPilot 2.0 [31], Analyst 1.5 with MIDAS [32], and Multiquant 2.0 software [33]. Peptide identification was confirmed using MASCOT 2.3. All raw mass spectrometry data were deposited in the Yale Protein Expression Database (YPED) [25,26] and are publicly available through the repository [27]. In our paper, we focus on the quality assessment and normalization of samples obtained from synaptic vesicle region (LP2). Among 1151 fragments, we select 822 fragments whose MS/MS peaks were detected and quantified for at least two runs.

### 2.3. Data Set 3: S. Pyogenes

*Streptococci. Pyogenes* (*S. Pyogenes*) is a highly contagious bacterium that causes many human diseases from strep throat and mild skin infections to necrotizing fasciitis and streptococcal toxic shock syndrome [34]. Teleman *et al.* designed an MRM assay for *S. Pyogenes* to compare the expression profiles of *Streptococci* strain SF370 grown in a pure Todd-Hewitt broth (TH) with and without 10% citrate treated human plasma (Skåne University Hospital, Sweden) [21]. Under each condition, 9 biological samples were grown. The assay monitors 161 peptides from 10 ribosomal proteins, 14 fatty acid synthesis pathway proteins, and 29 virulence associated proteins, including four house-keeping proteins, RS10_STRA1, RL22_STRP1, RL1_STRP1 and RS17_STRA1. Peak detection and quantification were performed by Anubis, which involves signal interference correction during peak area integration. A total of 138 peptides were quantified and the peptide-level peak intensity is reported by summing the transition areas. We use this data set to demonstrate that it may be more advantageous to perform normalization at the transition and peptide-level data. The data set is available at the Swestore repository [35].

### 2.4. Data Structure

**Figure 1:**
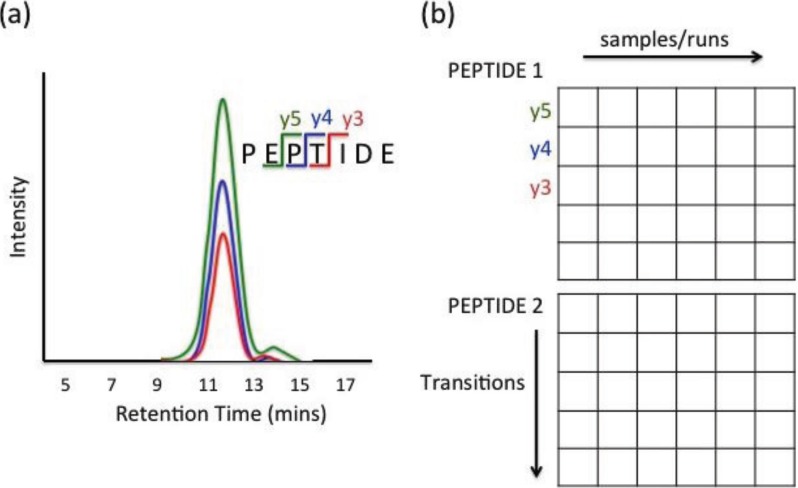
(**a**) Example of three Multiple Reaction Monitoring (MRM) extracted ion chromatograms from a single precursor ion (**b**) Data structure grouped by precursor ion (peptide) [36].

MRM data can be extracted through various ion chromatogram characteristics, such as retention time, signal-to-noise ratio, and peak abundance. Retention time is the amount of time elapsed from the injection of a sample into the chromatography system to the apex of the peak. Signal-to-noise ratio is a quality measure of each peak that provides the strength of intensity signal *versus* background noise. Peak area is widely used as the measure of abundance and is determined by the area under the intensity curve between peak start and end times. For analysis, each characteristic (e.g., area) can be structured in the form of a matrix. Figure 1 illustrates an example of data structure from PSD label-free experiment with six runs, where each peptide is measured by five transitions. Each row and column of the data matrix corresponds to a fragment and sample, respectively. Observation from one fragment and a given sample is recorded as an element of the matrix. The rows and columns of the matrix are often grouped according to precursor peptide sequences and biological samples.

## 3. Data Pre-Processing

### 3.1. Quality Assessment

#### 3.1.1. Adjusted Retention Time Deviation and Outlier Detection

**Figure 2:**
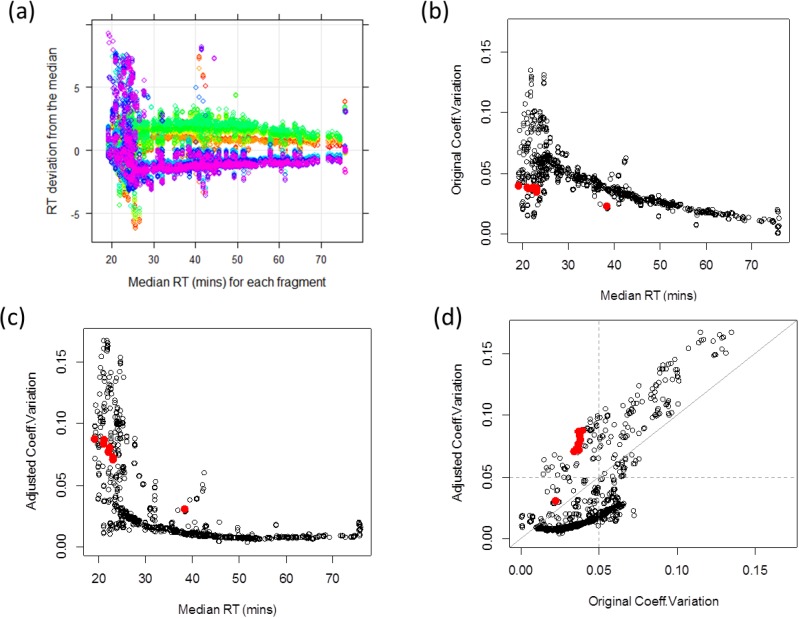
(**a**) Retention time deviations show global shifts between samples, (**b**) Coefficient of variation (CV) without retention time adjustment, (**c**) CV after adjustment, (**d**) Comparisons between the original and adjusted retention time CVs with diagonal grey line. Red dots correspond to nine transitions with original CV < 4% and adjusted CV > 7%.

Retention time of a given peptide is determined by its amino acid component. Since peaks from the same precursor should be identified at similar time points across MRM runs, observed retention time can be used to assess the accuracy of quantified peaks. One way to quantify the degree of variation is to calculate the coefficient of variation (CV) of the retention times across runs for each fragment. Empirical data suggest that systematic biases contribute to overall run-to-run variation, possibly due to the technical variations of chromatography. Figure 2a illustrates the retention time deviations of each run from transition-specific median retention time. Retention time deviations from each of 18 runs are labeled with different colors. Layers of colors indicate global retention time shifts across samples. For example, runs from Sample 3 (labeled in yellow to green) are almost always observed at later time points than runs from Sample 6 colored in navy to purple. Thus, it is important to adjust for such time shifts when comparing the observed retention times across multiple runs. One simple adjustment is to match the median retention time across MRM runs. Adjusted CV is smaller than the original CV if a transition’s retention time order is consistent with the global pattern. This is because the systematic bias inflates the original retention time variation, whereas these biases are accounted in the adjusted data. If a transition has an inconsistent retention time order across samples, the transition has higher adjusted CV than the original value. Figure 2b–d illustrates the original and adjusted retention times. Figure 2b shows that the original CV is large for transitions with early retention time and decreases over time. In contrast, adjusted CV corrects for the instrumental effect and provides more stable CV over time (Figure 2c). The 87.6% of transitions have larger original CV than the adjusted ones (Figure 2d, below diagonal line). Their median retention times cover almost the entire range, from 20 to 60 minutes. Red-labeled dots indicate nine transitions identified to be problematic from the adjusted retention time analysis. These transitions have small original CV’s (<4%) and large adjusted CV’s (>7%), *i.e.*, having inconsistent retention time order from all other transitions. In the PSD and CSP data, 1509 (89%) and 762 (92%) transitions exhibit the adjusted retention time CV greater than 5%.

#### 3.1.2. Peptide-Level Intensity Estimation via Robust Linear Regression and Measure of Outlying Peak Area

Robust MultiChip Analysis (RMA) is widely used for microarray analysis to summarize probe-level data into probeset-level intensity values [37]. It fits a linear model for a given probeset across samples by modeling the log2-transformed probe intensity as a function of the probe affinity effect and chip effect. These probe and chip effects are estimated by a robust approach, which evaluates a weight for each probe-level observation based on its concordance to the model. Use of this weight as a quality measure was first proposed for microarray gene expression data to assess the quality of each sample [38]. We briefly describe this robust model which can be applied to MRM data. For a given peptide, peak area is log2-transformed and modeled with the effects due to sample and transition. The model is shown in Equation (1), where *y_ij_* denotes log2-scaled peak area from transition i and sample j, *μ* is the average log2 peak area, *α_i_* is the effect of the i-th transition, and *μ_j_* is the effect of the j-th run. For identifiability, sum of all *α_i_* is set to be zero.

*y_ij_* = *α_i_* + *μ_j_* + *ϵ_ij_*, *ϵ_ij_* ~ *i.i. d N* (0,*σ*^2^) (1)

**Figure 3:**
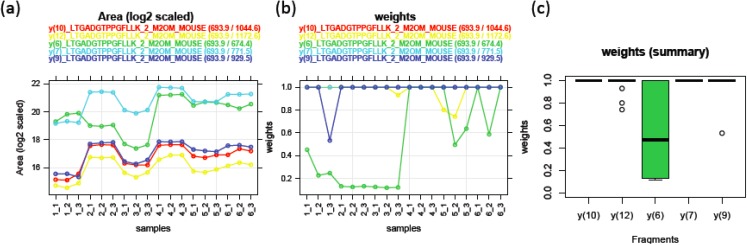
(**a**) Log2 transformed peak areas for five MRM transitions of M2OM peptide LTGADGTPPGFLLK (postsynaptic density (PSD) assay (n = 18)). Peak areas from the same MRM transition are connected with a unique color label. (**b**) Weight as a measure of outlying behavior of each peak. (The weights of y(10), y(12), and y(7) are mostly 1, thus the corresponding red, yellow, and light blue curves are overlapping with the blue curve for y(9).) (**c**) Distribution of weights for each transition.

In MRM experiments, transition-level peak intensities from a fixed peptide depend on a fragmentation pattern during the precursor collision stage (Q2). A pair of transitions fragmented from the same peptide should have proportional intensities with one another. Equation (1) takes this pattern into account by using the additive transition effect (*α_i_*) after log-transformation. For example, 2^*α*_1_−*α*_2_^ denotes the peak intensity ratio between transition 1 and transition 2. Model parameters (*μ*, *α_i_*, *β_j_*, *σ*^2^) can be estimated by robust linear regression. It is an attractive alternative to the ordinary linear regression model when there are concerns about potential outliers. While the ordinary method estimates the parameters by minimizing sum of squared residuals, the robust approach calculates a weight *w_ij_* for each observation according to the magnitude of its residual and then, tries to minimize the sum of weighted squared residuals. Iteratively reweighted lease-squares (IRLS) is adopted to estimate the model parameters and weights alternately until convergence. The weight has a value between 0 and 1. A larger weight for a peak implies a more consistent peak behavior with regards to the overall pattern. It can be used as an individual peak quality measure. Figure 3 shows an example of the robust model fitted for PSD data. Figure 3a displays log2 transformed peak area from five transitions fragmented from a doubly-charged peptide LTGADGTPPGFLLK of M2OM protein. Peak intensities from different transitions are labeled with different colors and linked across 18 runs. Four transitions (y(10), red; y(12), yellow; y(7), light blue; y(9), blue) exhibit parallel patterns indicating the area ratio among these transitions are consistent across all the runs. On the other hand, y(6) transition (PGFLLK, green line) shows non-parallel relationship with the rest of transitions. For example, y(6) peak areas are slightly higher than y(7) peak areas for PSD1 runs, while they are much lower for PSD2 and 3 runs. This inconsistency in y(6) transition is well captured in its weights as shown in Figure 3b. The majority of weights estimated for y(6) peaks are around 0.5 or lower, indicating that many of y(6) intensities are outlying. In contrast, most of the weights from other transitions are 1.

#### 3.1.3. Use of Robust Model to Measure Inconsistent MRM Transitions

**Figure 4:**
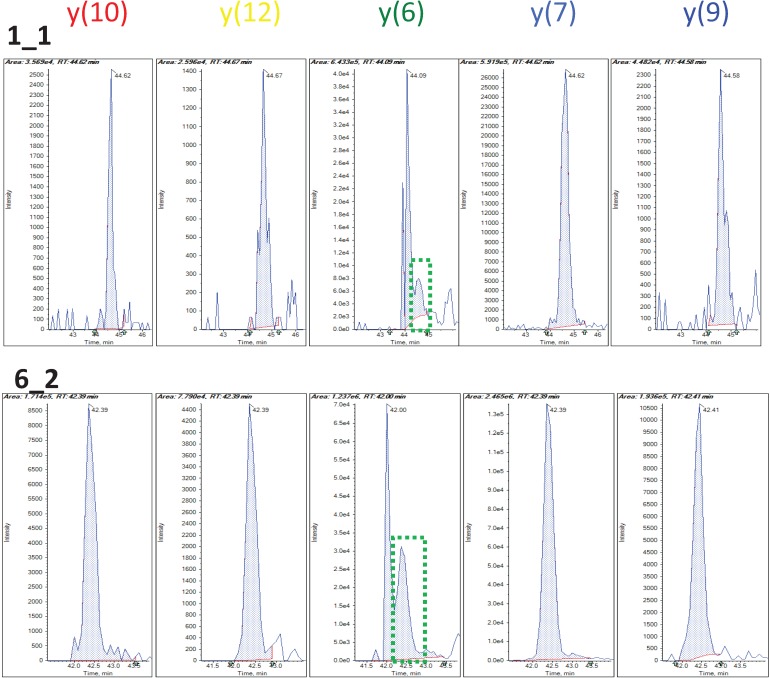
Peak shapes from two runs in PSD data from the same peptide as in Figure 2. Middle panel shows the peaks of a problematic transition by median weight by robust regression. Green dotted boxes show correct peak candidates.

**Figure 5:**
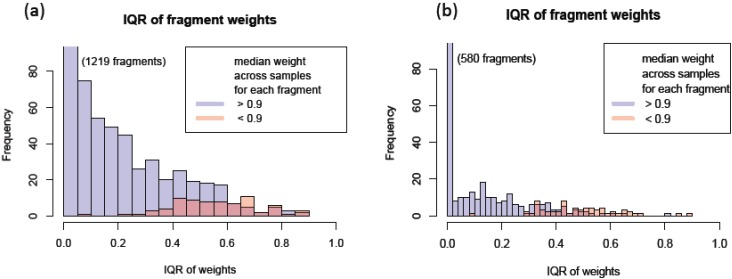
Interquartile region of transition peak weights for transitions with median weight >0.9 and <0.9 for (**a**) PSD data and (**b**) Cysteine string protein (CSP) data.

In addition to using the robust regression model to infer the quality of individual peak, we can also use this model to assess the consistency among individual fragments. One way to measure the transition’s overall accuracy and consistency is to look at the median weight across all the MRM runs. Another characteristic to examine is the spread of weight estimates. The Inter-Quartile Region (IQR) is defined as the range between the 1st quartile (25th percentile) and the 3rd quartile (75th percentile) and it can be used as a measure of variation in the weights. These two characteristics can easily be displayed by using box plots as in Figure 3c. The median weight is illustrated with a band inside the box and the height of the box indicates the IQR. Qualified transitions should have high median weight and small IQR. It graphically summarizes that many peak intensities from y(6) of this selected peptide have overall low and varying weights, while all other transitions have weights close to 1. Figure 4 shows the actual peak shapes from five transitions from this peptide from two runs (PSD1 technical run 1 and PSD6 run 2). The y(6) peaks from these two runs have low weights of 0.45 and 0.59, respectively. It is clear from Figure 4 that the peaks from this transition (middle panel) have signal interference and peak detection was not accurate. We detected 1622 (95%) and 756 (92%) fragments as having the large median weight greater than 0.9 from the PSD and CSP experiments, respectively. Figure 5a,b show the distribution of the IQR of the transition weight according to its median weights classified at 0.9. The IQR of these transitions is overall higher, greater than 0.4 (red-colored distribution) than the region of good quality transitions.

#### 3.1.4. Use of Robust Model to Assess the Sample Quality

**Figure 6:**
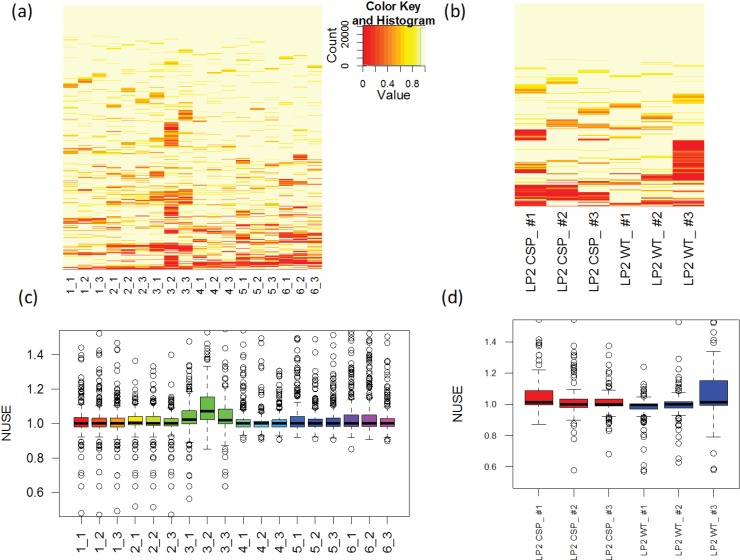
Individual weights are graphically summarized and Normalized unscaled standard error (NUSE) distributions are plotted for (**a**), (**c**) PSD and (**b**), (**d**) CSP data, respectively.

Figure 6a,b display the individual weights across all the transitions and all the runs in the PSD and CSP data, respectively. Transitions are re-arranged by hierarchical clustering. One can see that many transitions in some runs have low weights compared to other runs. For example, PSD3 technical run 2 (column 3_2 in Figure 5c) has 482 transitions (32%) with weight less than 0.9. In this section, we discuss the use of regression weight as a measure of sample quality. Let us revisit the regression model and weight estimation in Section 3.1.2. The quality of a given peptide in each run (*j*) can be evaluated as 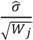, where 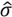 is the estimated standard deviation of error term (Equation (1)) and *W_j_* = Σ_*i*_*w_ij_* is a total transition weight [38]. However, this estimate depends on the standard deviation estimate 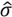 and does not consider heterogeneity among peptides. Furthermore, it does not take into account the variation in the number of transitions per peptide or the presence of poor quality fragments. Normalized unscaled standard error (NUSE) was proposed as a measure of overall sample quality using the weights [38]. Briefly, it first replaces 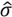 by 1. Then, the authors suggested to normalize the value through dividing it by its median across all samples, *i.e.*,

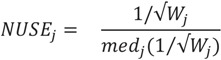


The NUSE value of a specific peptide varies around one and sample quality can be stated as a summary of the NUSE values across all the peptides. Similarly with the fragment quality assessment in the previous section, box plot gives a nice visualization on the sample characteristics. Numerically, median NUSE values or IQR provide an overall summary of the overall sample quality. This is illustrated through Figure 6c,d that show our example data sets. In PSD experiment, sample 3 run 2 shows low quality with its median NUSE value over 1.05 (>5%), while all other runs have the values between 0.99 and 1.02. In CSP data, all six runs have their median NUSE below 1.5%. However, large fluctuation of the NUSE values in KO replicate 1 and WT replicate 3 (>10%) suggests quality issues in those runs.

### 3.2. Data Normalization

#### 3.2.1. Source of Experimental Artifacts

**Figure 7:**
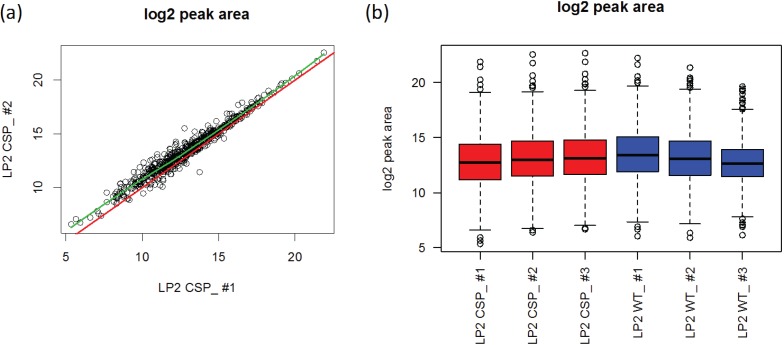
Comparison between two technical replicates from CSP knockout (KO) sample (**a**) and overall peak area distribution among CSP KO and wild type (WT) samples (**b**).

An effective normalization method aims to minimize non-biological, systematic variations across samples so as to make the samples more comparable. The unwanted factors include differences in the total protein amount loaded into the instrument, instrumental variations, instrument settings, and peak modeling and extraction settings. For example, the study of CSP assay reveals a global change in the peak signals even between technical runs, that is, peak area from technical run 1 is slightly higher than that from run 2 of CSP KO sample (Figure 7a). The overall distribution of log scaled peak area also fluctuates as shown in Figure 7b.

#### 3.2.2. Using All the Transitions in the Data

To adjust for systematic variations among samples, various approaches have been proposed for gene expression microarray experiments. One type of methods utilizes all the features in the data set for normalization [15,17,18,39]. These methods assume that (1) intensity distributions are similar across samples, (2) the majority of features have consistent intensities, without being altered by experimental states, and (3) the proportions of over- and under-expressed features are similar. The simplest way is to assume a constant median and normalize the data by subtracting the sample-specific median. This approach is also commonly used in MRM analysis [20]. Although this approach makes the overall median intensities identical, it does not consider intensity variation across features. For example, WT run 3 has a relatively smaller IQR than the WT run 2 as shown Figure 7.

Quantile normalization is one popular approach for gene expression normalization, especially for the data generated by Affymetrix oligonucleotide arrays [15]. It is designed to make the distribution of intensities to be identical across samples. The quantile normalization procedure works as follows. First, intensities from each sample are arranged from the smallest to the largest. The average is taken across samples, for example, the average of the collection of the smallest intensities or the average of the collection of the largest intensities. Then, the original intensities are replaced by the averaged values and re-arranged in the original order. Through this procedure, intensity distribution becomes identical across all the samples.

Cyclic loess is an alternative approach designed to remove intensity-dependent pattern between a pair of samples [17]. Loess normalization was first applied to two-color microarray experiments [39]. It fits a locally weighted smoothing curve to an MA plot, where the log2-difference (M) between two samples of each probeset is plotted on y-axis and the log2-average is plotted on x-axis (A). Then, the adjustment is made by subtracting the fitted loess value from the original M value. Cyclic loess is an extension for experiments having more than two samples to be normalized. The adjustment is carried out by performing the loess normalization for a pair of samples and repeating the procedure for all possible distinct pairs. These steps are performed in an iterative manner until convergence. The cyclic loess approach demonstrated better performance than other approaches including quantile or linear baseline normalization, in reducing variation among replicated samples for LC-MS based metabolomics experiment [40].

#### 3.2.3. Using a Subset of the Data

Normalization using all the data performs well for high-throughput experiments, where tens of thousands of features are simultaneously monitored and most of them are expected not to be altered across different cellular states. On the other hand, MRM studies examine a pre-defined subset of proteins/peptides that are likely to be involved (and altered) in these studies. If the treatment effects are expected to cause changes of expression in the set predominantly in one direction, the global normalization strategies that we discussed above may introduce artifacts in the data or even remove the treatment effects of interest. To address this limitation, a number of subset-based approaches have been proposed, mostly in the context of normalization of miRNA microarrays and qRT-PCR results, where only a few hundred miRNAs are profiled instead of monitoring all miRNA present in the model organism [41,42].

An alternative strategy is to use a set of control proteins, peptides, or transitions. A good control set should have stable intensity in response to treatments of interest. Normalization using a subset of the fragments involves three steps as follows: determination of control features, normalization with the control features, and application of the same adjustment to all the features in the data. One way to select the set of control features is to introduce internal standard (IS) peptides. For example, an MRM study of schizophrenia utilized spiked enolase peptides [43]. In our PSD data set, four IS peptides were added (3 transitions/peptide) before the sample being run by LC-MS-MS experiments. These peptides can be assumed to be non-differentially expressed. However, subset-based normalization using known proteins or peptides have several drawbacks because the number of pre-determined IS peptides is often small and their intensities do not cover the whole range of intensity levels in the data. Furthermore, the abundance of IS peptides can exhibit natural variation. Another limitation is that the abundance of IS peptides does not capture the variation arisen during sample preparation but instrumental variation since they are added right prior to the MS run. Another approach is to use proteins known to be consistent across treatments. For example normalization of *S. Pyogenes* MRM data was performed by using peptides from four housekeeping proteins [21]. However, it is only feasible when the house keeping proteins are known for the model organism and experimental states. Once a subset is defined, normalization can be applied to the subset by calculating mean or median factor [21,41]. Then, the normalization is extended for all the features in the data by subtracting the scaling factors. In the following section, we report the result of median-based approach. The use of mean-based scaling factor led to similar performance to the median-based method.

Invariant set normalization is an alternative approach to selecting a collection of features to be used for data normalization [44]. This approach was originally developed for two-color microarrays, where one channel measures sample intensity and the other channel measures reference intensity. In the multi-sample normalization, one can choose a baseline array or mean or median gene expression as a reference. Expression of each array is then compared with the reference. Briefly, invariant genes are defined as those having consistent ranking among channels or samples. Rank-invariant set is determined by an iterative method, which classifies genes as non-differentially expressed if the rank difference is less than a pre-specified cutoff [44]. Then, the normalization is performed by interpolating the intensity from the loess curve. In our analysis, we used a set of transition-specific median as a reference.

#### 3.2.4. Comparison of the Normalization Performance

We compared the performance of 5 normalization approaches: (1) median adjustment, (2) quantile normalization, (3) cyclic loess, (4) median adjustment using internal standard peptides, and (5) invariant set normalization. Among these methods, methods (1), (2), and (3) are based on the global intensity profiles, whereas methods (4) and (5) are based on a subset of proteins or peptides. The normalization of PSD and CSP data were performed at transition-level and *S. Pyogene* data at peptide-level.

First, we examined the reduction of coefficient of variation (CV) after normalization. Since the PSD samples show heterogeneity due to the preparation protocol and CSP and *S. Pyogene* data were collected from two different treatment groups, CV is evaluated by taking this grouping structure into account. Briefly, we computed the sum of squared residuals by fitting a regression model with the group variable for a fixed transition:

*y_kj_* = *μ* + *γ_k_* + *ϵ_ij_*, *ϵ_ij_* ~ *i.i. d N* (0,*σ*^2^)

where k = 1 or 2 indicates group and j = 1,2, .., J for replicated samples. Group specific mean is captured by *γ_i_*. The unbiased estimator of the experimental noise (*σ*^2^) is obtained by mean squared error (MSE), which divides the sum of squared residuals by its degrees of freedom. A better normalization approach should have a lower MSE, and reduces non-biological variations among replicated samples. The CV is obtained as a ratio of root MSE and the average (log2) peak area for each transition or peptide. Median CV for each data set is described in Table 1 across multiple normalization methods. It reveals that any normalization approaches assist reducing the CV.

**Table 1:** Median coefficient of variation for the five normalization methods considered (with median absolute deviation).

Data Set	Original	Global Median	Quantile	Cyclic Loess	IS.Median	Invariant Set
PSD	4.18% (1.96%)	2.15% (1.41%)	1.97% (1.12%)	2.12% (1.39%)	3.09% (1.51%)	1.99% (1.29%)
CSP	4.04% (2.82%)	3.47% (2.75%)	2.85% (2.03%)	3.26% (2.16%)	NA	3.29% (2.43%)
*S. Pyogene*	3.05% (2.78%)	3.47% (2.84%)	2.34% (2.02%)	2.85% (3.15%)	3.12% (3.32%)	2.69% (2.74%)

**Figure 8:**
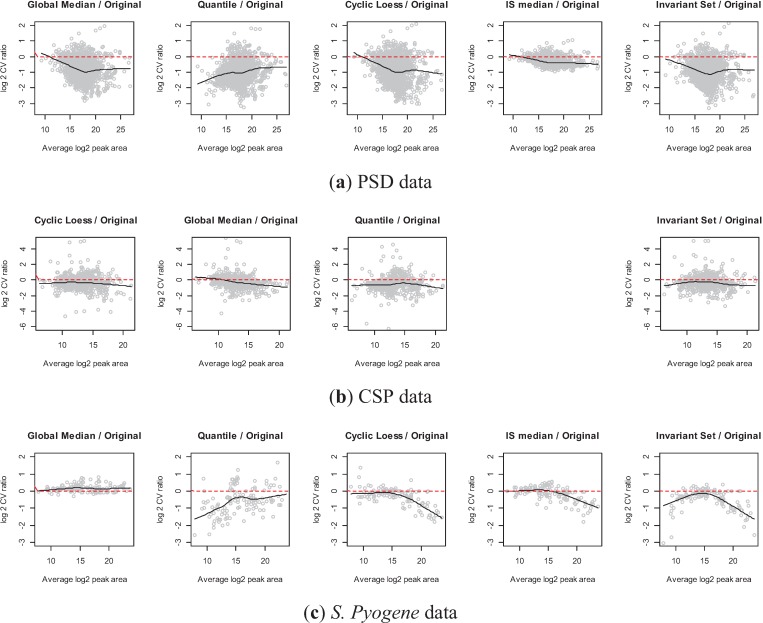
Comparisons of each normalization method *vs.* original data ((**a**) PSD, (**b**) CSP, and (**c**) *S. Pyogene*). Each plot shows the log2 ratio of CVs on y-axis and the average peak area on x-axis. Black line is a smoothing curve illustrating the overall pattern and the red dashed line indicates equal CV between normalized and original data.

Figure 8 shows the log2 scaled CV ratio between each normalization method and the original data on y-axis along with the average peak area on x-axis. Negative values on y-axis indicate that the transition has reduced variance due to normalization. In PSD data (Figure 8a), all global and sub-set based approaches led to reduced within-sample variation. Especially, the CV ratio decreased for transitions with high average intensities, except for the quantile normalization for PSD data. It implies that one may gain considerable variance reduction for highly abundant transitions. On the other hand, the effect of variance reduction was minor for low-abundant transitions. It can be due to the few transitions with low-abundant peak areas, which introduce an artifact in smooth line fit. Another possible reason is that a low abundant transition is more likely to have undetected or low quality peaks. We observe large within sample variation for such transitions and the magnitude of decreased variation by normalization approach was small. The CSP experiment shows slightly decreased variation by any normalization approaches. Two subset-based normalization approaches for *S. Pyogene* data illustrate that the performance highly depends on the average log2 peak area. In the IS.Median approach, a subset of pre-specified proteins was used [34]. These proteins have an overall high peak area (>18 in log2 scale), leading better performance on high intensity peptides. Invariant set normalization has a slight advantage over the pre-defined set as it selected peptides covering overall intensity range.

**Figure 9:**
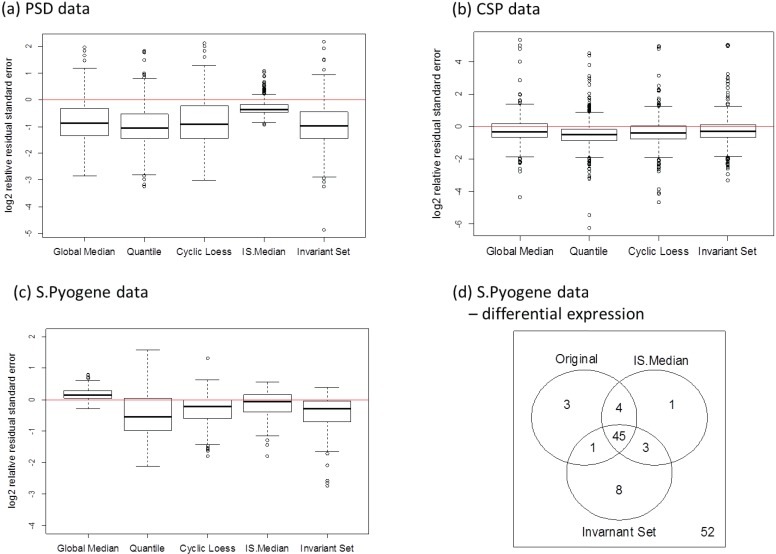
Differences between the within-group deviation between normalized and original data for (**a**) PSD, (**b**) CSP, and (**c**) *S. Pyogene* data along with the two-group comparison result at 5% false discovery rate (**d**).

Our second approach is to examine the magnitude of variance estimates directly instead of CV. The unbiased estimator of the experimental noise (*σ*^2^) is obtained by MSE. The ratio of the MSE is evaluated from normalized data to the MSE from the original data. The ratio is calculated for each transition and the ratio distributions are shown in Figure 9a–c. The within-group variance was reduced substantially especially when the data were collected over different biological replicates (a, c). Quantile and invariant set normalization performed better than the other methods. The use of housekeeping (c) proteins is also advantageous over the use of spiked-in peptides (a). Additionally, we performed a differential expression analysis for the *S. Pyogene* data set for the comparison between normal and human plasma growth conditions. The Welch’s t-test was used to evaluate the peptide alteration and a *p*-value cutoff of 0.05 was used to determine significant changes. Subset-based normalization using house-keeping peptides detected the same number of differentially expressed peptides with the original data, but it was able to identify significant alteration of both D-alanine—poly(phosphoribitol) ligase subunit 1 and 2 (DLTA and DLTC) which agree with previous studies [21,45]. Rank-invariant set normalization showed improved power to identify differentially expressed peptides compared to the original data analysis.

## 4. Conclusions

In this paper, we have discussed pre-processing approaches for label-free MRM data sets. We considered the assessments of transition and sample quality and data normalization. For quality assessment, we suggest to examine the consistency of retention time and peak area across all the samples in the experiment. The first approach is to focus on the retention time and select transitions with large retention time variation. By adjusting for global retention time variation, it provides more robust detections of problematic transitions with mis-identified peaks. The second approach aims to evaluate inconsistent MRM peaks by decomposing the peak area with transition effect and sample‑specific variation. Robust linear regression evaluates weights for individual peak which can be used as evidence for outlying quantitation. By summarizing the weights at the transition- or sample-level, one may be able to identify inconsistent transition or problematic MRM samples. The distinctive feature of this quality assessment tool lies in its data-driven nature. We note that other methods have been proposed for quality assessment, such as to utilize spectral library intensity (Skyline), peak intensities from heavy labeled transitions (AuDIT), or peak characteristics from decoy transitions (mProphet). These methods require specific assay development or MS/MS peak monitoring scheme. In contrast, we believe that our pipeline is advantageous in that it does not require extra information and borrows information across samples. Our approaches model retention time or peak area across all the samples processed together in a label-free MRM experiment, and are more generally applicable. This pipeline can be used along with the Skyline’s label-free function or independently for the MRM data quantified by other approaches.

For normalization, we have compared the performance of five normalization methods through their applications to three MRM data sets. Our results suggest that these normalization approaches may reduce within-group variation, and increase power to identify differentially expressed peptides or proteins. In particular, in the case of *S. Pyogene* data, the use of house-keeping proteins was advantageous in reducing biological variations in the MRM experiment. Although internal standard peptides can also be utilized in data normalization, the results from the PSD data suggest that it may be less appropriate as those peptides may not capture all technical artifacts in the MS instrumental variation. We note that the use of such house-keeping proteins or peptides is only suitable if those proteins are known and not altered by specific experimental conditions. In the absence of such proteins, our results suggest that quantile normalization or rank-invariant set normalization may be preferred approaches for data normalization.

Workflow discussed in this paper provides automated selection of high quality transitions and MRM runs followed by adjusting for experimental artifacts. We believe that this pipeline can assist MRM assay development, data collection, and downstream analysis. The R package for this pipeline, MSq, is available online [46].
